# Treatment of L5 - S1 intervertebral disc herniation with posterior percutaneous full-endoscopic discectomy by grafting tubes at various positions via an interlaminar approach

**DOI:** 10.1186/s12893-019-0589-2

**Published:** 2019-08-28

**Authors:** Weijun Kong, Taiyong Chen, Sheng Ye, Fujun Wu, Yueming Song

**Affiliations:** 1 Department of Orthopedic Surgery, West China Hospital, Sichuan University, No. 37 GuoXue Road, Chengdu, 610041 Sichuan China; 2 Department of Spine Surgery, The Affiliated Hospital of Zunyi Medical University, No. 149 DaLian Road, Zunyi, 563000 Guizhou China

**Keywords:** Lumbar disc herniation, Percutaneous, Full-endoscopy, Interlaminar space, grafting tube

## Abstract

**Background:**

Depending on the location of the herniated disc at the shoulder, axilla, or ventral side of the compression nerve root, various puncture sites and channel entrances were selected so that the goal of targeted removal of the herniated disc could be achieved by a full-endoscopic technique. Achieving good clinical therapeutic efficacy through the natural gap of bones can maximally avoid related access complications, and the necessary techniques and relevant anatomical factors were analyzed.

**Methods:**

Between August 2012 and August 2014, 98 patients with L5 - S1 intervertebral disc herniation were treated with posterior percutaneous full-endoscopic discectomy (PPFED) by grafting tubes at various positions via the interlaminar approach. The visual analog scale (VAS) and the Oswestry disability index (ODI) were used to assess the patients’ back and leg pain and the improvements in daily function, and the modified Macnab standard was used to evaluate the treatment efficacy.

**Results:**

All 98 patients successfully completed the surgery, 84 patients got out of bed and walked on the first postoperative day, and 14 patients got out of bed and walked on the second postoperative day. The preoperative ODI (56.032 ± 3.625) was significantly higher than the ODI score (8.147 ± 1.398) (F = 5343.054, *P* ≤ 0.001) 48 months after surgery. The preoperative VAS score (7.193 ± 0.875) was significantly higher than the postoperative VAS score (0.914 ± 0.500 points) (F = 1656.173, P ≤ 0.001). The differences in ODI and VAS scores before and after surgery were statistically significant (*P* < 0.05). Follow-up was conducted 1, 6, 12 and 48 months postoperatively, and the modified Macnab standard was used during the last follow-up to evaluate the efficacy: 67 cases were excellent, 20 cases were good, 7 cases were fair, and 0 cases were poor; the proportion of excellent and good cases was 92.6%.

**Conclusions:**

The treatment of L5 - S1 intervertebral disc herniation with PPFED by grafting tubes at various positions via an interlaminar approach is a safe, effective, and minimally invasive surgical method. Reaching the location of a disc herniation directly through the natural gap in the bones can maximally avoid collateral injury from spine surgery.

**Trial registration:**

The registration number of this clinical study is ChiCTR1800014588; it has been retrospectively registered with a registration date of 05/01/2018.

## Background

Most patients with lumbar disc herniation can achieve good treatment results through conservative treatment; only a small portion of patients require surgical treatment [1]. Traditional interlaminar fenestration and intervertebral disc removal together with interbody fusion still comprise a routine surgery for the treatment of lumbar disc herniation (LDH) [2]. To reduce surgical trauma and the occurrence of related iatrogenic complications and at the same time accurately remove the herniated disc tissue, minimally invasive techniques have gradually been developed in spinal surgery, including chemonucleolysis, percutaneous intervertebral disc resection, resection of the nucleus pulposus, minimally invasive intervertebral disc resection, percutaneous transforaminal endoscopic discectomy, and microscope-assisted discectomy [2–4]. With the rapid development and continuing improvement of endoscopic, optical, and channel technology, spinal endoscopy has become the prime surgical method for LDH treatment due to its clear field, minimal trauma, targeted resection of protruding lesions, capacity to prevent injuries to paraspinal muscles, lamina and other structures, and significant reduction of complications related to the early return of patients to society and work; at the same time, it achieves superior cosmetic effects compared with open surgery. The posterolateral transforaminal endoscopic approach is a more widely used approach; however, for patients with L5 - S1 intervertebral disc herniation, the posterolateral approach is limited due to the high iliac crest, the narrow interlaminar space and nerve root foramen, the hypertrophic transverse process of L5, hyperplasia of the articular process, and other anatomical and degenerative factors in most patients [5]. The interlaminar space of L5 - S1 and the natural bone gap of the vertebral lamina are mainly on the same axial image; therefore, resection of protruding intervertebral disc tissue through the intervertebral laminar space under a full-endoscope method naturally becomes an ideal approach [6]. From August 2012 to August 2014, we used posterior percutaneous full-endoscopic discectomy (PPFED) through the interlaminar approach to treat 98 patients with L5 - S1 intervertebral disc herniation. The follow-up period was longer than four years, and satisfactory treatment results were achieved, as reported below.

## Methods

### General data

A total of 98 patients who underwent PPFED for L5 - S1 intervertebral disc herniation at the Department of Spine Surgery, The Affiliated Hospital of Zunyi Medical University from August 2012 and August 2014 were reviewed. The patients included 53 males and 45 females; their age ranged from 23.2 to 68.5 years, with an average of 51.3 years. Shoulder protrusion occurred in 49 cases, axillary protrusion was present in 31 cases, and 18 cases had ventral nerve root protrusion. All patients had lower back pain, typical radiating pain or numbness in a unilateral lower extremity, and a positive sign in a straight leg raise test. The disease course was 1–26 months, with an average of 4.6 months. The preoperative visual analog scale (VAS) and the Oswestry disability index (ODI) scores of these patients are shown in Table 1.

**Table 1:** Comparison of preoperative and postoperative VAS and ODI scores (*n* = 94)

Score	Pre-op.	Post-op. 1 M.	Post-op. 6 M.	Post-op. 12 M.	Post-op. 48 M.	*Pillai’s Trace*	*F*	P
VAS	7.193 ± 0.875	1.860 ± 0.509	1.449 ± 0.474	0.925 ± 0.650	0.914 ± 0.500	0.982	1656.173	0.001
ODI	56.032 ± 3.625	9.198 ± 1.265	8.576 ± 1.230	8.256 ± 2.360	8.147 ± 1.398	0.994	5343.054	0.001

(Excluding one case of repeated calculation and two cases of re-operation, 94 cases were actually analyzed. *Pre-op* Preoperative, *Post-op* Postoperative, *M* months)

The case selection criteria were as follows: ① the symptoms were not improved after three weeks of conservative treatment for unilateral sciatica; ② the patient was positive for the straight leg raise test; ③ CT and MRI examination suggested single-segment L5 - S1 posterolateral intervertebral disc herniation that was consistent with the signs; and ④ adequate communication with patients who voluntarily chose treatment with endoscopic surgery. The exclusion criteria were: ① patients with central, foraminal or extreme lateral intervertebral disc herniation of L5 - S1 segments; ② patients who had undergone previous open surgery for the same segment on the same side; ③ patients with combined infection, tumor, and fracture; and ④ patients in whom the same segment was accompanied by spinal slippage and instability.

**Fig. 1:**
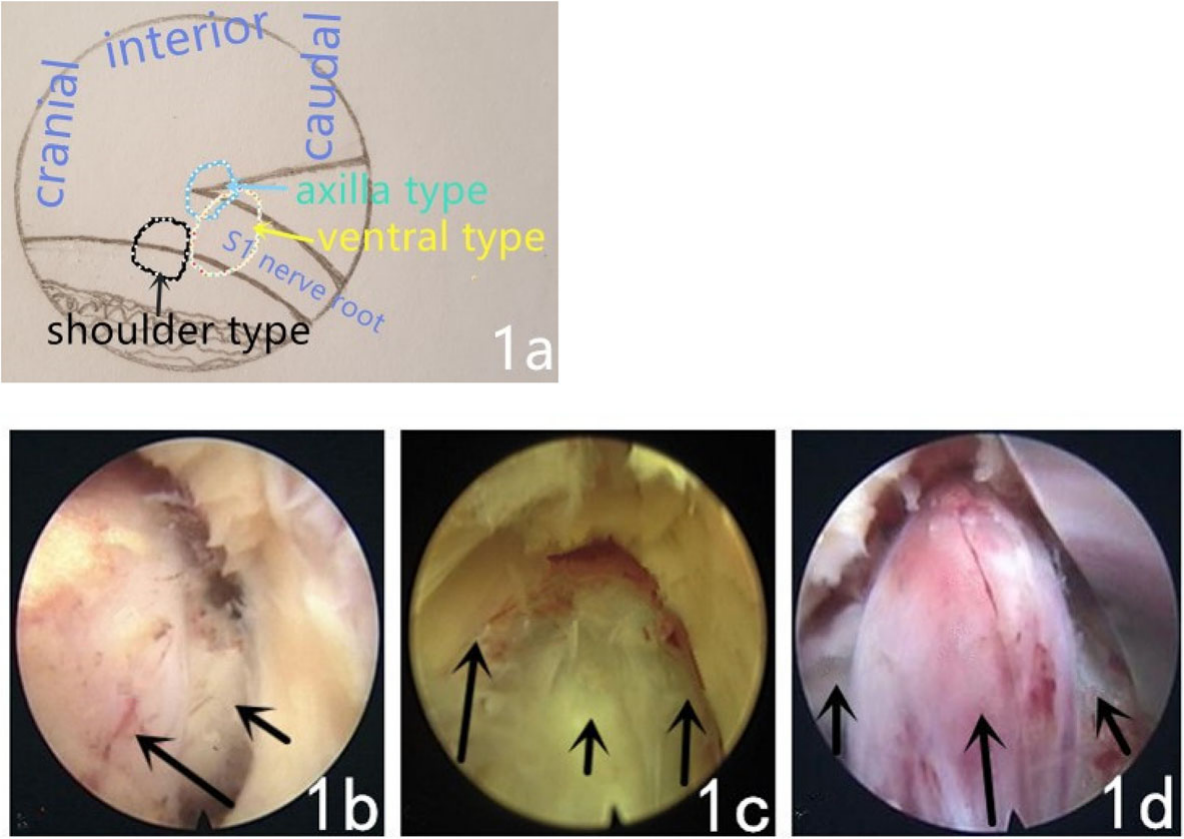
**a**. A schematic view of the protrusion of disc. **b**. The disc can be seen intraoperatively protruding at the shoulder of the nerve root (the long arrow shows the nerve root, and the short arrow shows the protruding disc tissue). **c**. The disc can be seen intraoperatively protruding at the axilla of the nerve root (the long arrow shows the dura sac, the medium arrow shows the nerve root, and the short arrow shows the protruding disc tissue). **d**. The disc can be seen intraoperatively protruding at the ventral side of the nerve root (the long arrow shows the nerve root, and the short arrow shows the protruding disc tissue)

This study was approved by the Zunyi Medical College Institutional Review Board. Anteroposterior and lateral X-rays of the lumbar spine and CT and MRI of the lumbar intervertebral disc were completed preoperatively and used to evaluate the intervertebral approach. At the same time, a preliminary judgment of the relationship between the position of the herniated disc and the S1 nerve root was conducted (Fig. 1). Depending on whether the herniated disc was located at the shoulder (in the axial image, the herniated disc is located posterolateral, and in the sagittal view, the herniated disc is located at the lower margin of the posterior L5 vertebra), axillary (in the axial image, the herniated disc is located posterolateral, and in the sagittal view, the herniated disc is located at the posterior upper edge of the S1 vertebra), or on the ventral side of the nerve root (in the axial image, the herniated disc is located posterolateral, and in the sagittal image, the herniated disc is on the same axis as the L5 - S1 intervertebral space), various puncture sites and channel entrances were selected.

### Surgery

**Fig. 2:**
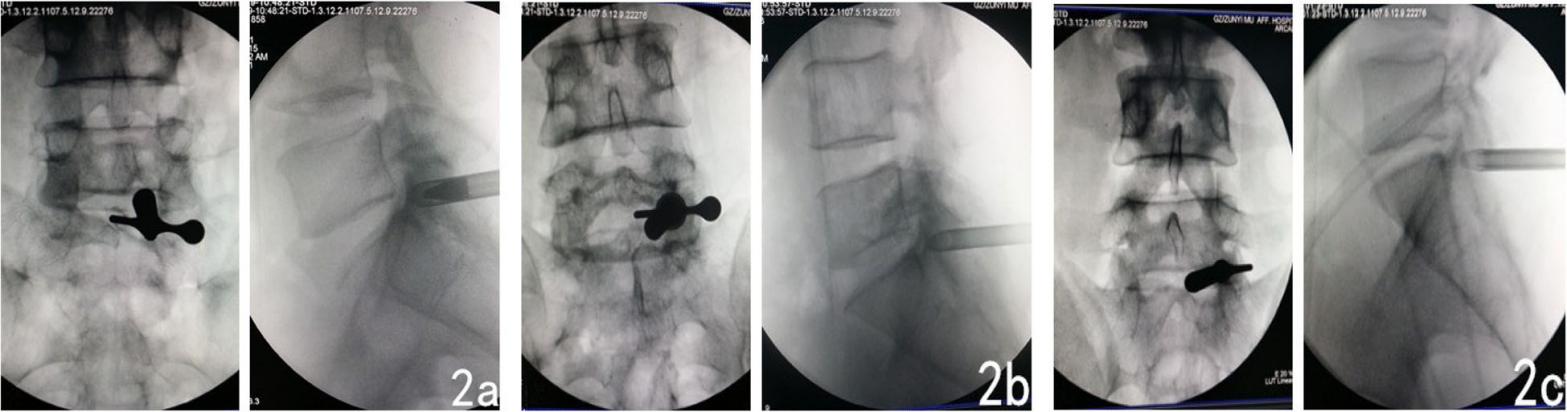
Anteroposterior X-ray image of intraoperative cannulas: **a**. shoulder type; **b**. ventral side type; **c**. axillary type

**Fig. 3:**
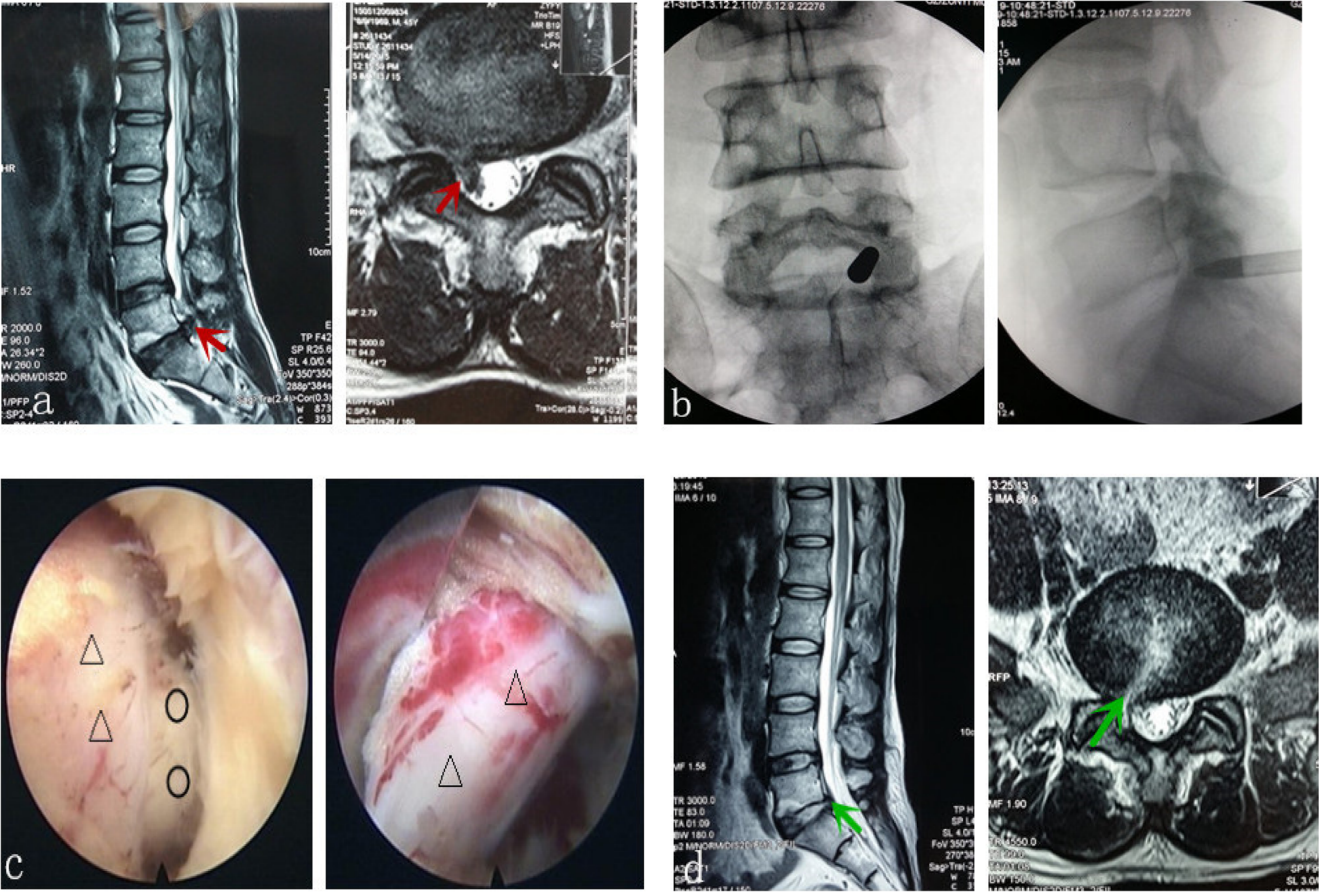
Male 45 years old, S1 nerve root shoulder-type disc herniation (shoulder type). **a**. The preoperative magnetic resonance suggested an obvious prolapse of the disc. **b**. Image of the inserted expansion cannula. **c**. Disc herniation and the relaxation of the nerve root after decompression were observed intraoperatively. **d**. A postoperative review by magnetic resonance shows the complete removal of the protruding disc

A thousand-grade purity laminar flow operation room was used. The choice of anesthetic method was sufficiently discussed with the patients and their relatives. A total of 91 patients chose continuous epidural anesthesia, and 7 patients chose general anesthesia. The patient assumed a prone position on a carbon-fiber operating bed that permitted taking X-ray images. The upper chest and the bilateral iliac crest were padded with a soft pillow so that the abdomen was suspended; this reduced venous return pressure in the spinal canal and reduced intraoperative bleeding. The patient’s hips and knees were flexed so that the spine protruded rearward, facilitating the opening of the interlaminar space and the placement of a working cannula. The internal margin line of the S1 pedicle on the symptomatic side was marked, and using accurate positioning according to the Ferguson X-ray perspective, the puncture spot was marked on the skin. Strict surgical area disinfection was performed, a sterile application was used to cover the surgical area, and precautions were taken to prevent the water used for rinsing from wetting the sterile towel. A sterile dressing sleeve was used to wrap the C-arm to prevent contamination of the surgical area when taking the X-ray. The center of the interlaminar space of the ipsilateral side was marked according to the X-ray, and 4 quadrants were divided according to this center. The shoulder-type puncture site was located in the upper-outer quadrant (Fig. 2a), and the puncture guide needle was inserted percutaneously. The anteroposterior image of the point of the needle was located in the upper-outer quadrant immediately adjacent to the inner lower edge of the inferior articular process of L5, and the lateral image of the point of the needle was at the posterior wall of the spinal canal and at the center of the L5 endplate axially. With the guide needle as the center, a skin incision approximately 7 mm in length was made, and the expansion cannula was inserted into the articular process along the guide needle; the guide needle was then pulled out, and the cannula was pushed with steady force past the ligamentum flavum to enter the spinal canal. Since the tip of the expansion cannula is round and dull, it will not damage the dural sac or the nerve root when extreme force is avoided. A working channel was inserted into the posterior wall of the spinal canal along the expansion cannula, and the operation system was sent in. Under the endoscope, the epidural fat, the nerve root, the space of the disc flavum ligament, the herniated disc tissue, and other spinal canal structures can be revealed. Radiofrequency was used to treat the epidural fat, and the inner ligamentum flavum was taken out appropriately to expand the space of the disc flavum ligament; following this, the protruding disc tissue that was compressing the nerve root could be fully exposed. When necessary, the tip of the working channel was rotated appropriately toward the spinal canal to avoid the nerve root, and the protruding disc tissue was then safely and completely removed (Fig. 3). The ventral side-type puncture site (Fig. 2b) directly faced the nerve root; its anteroposterior image was in the center of the interlaminar space, while its lateral image was at the upper edge of the vertebral endplate. The tip of the working cannula directly faced the nerve root; the direction of the cannula was adjusted toward the head or the tail side, the space of the disc flavum ligament was expanded, and the protruding disc was removed from the shoulder. The disc tissue protruding from the axilla also needed to be probed and removed to achieve disc resection around the nerve root and to eliminate the compression of the nerve root. The axilla-type puncture site was located in the lower inner quadrant of the interlaminar space (Fig. 2c). The puncture guide needle was inserted percutaneously; its anteroposterior image was at the midpoint of the line connecting the lateral margin of the spinous process and the center of the interlaminar space and the upper edge of the S1 lamina, while its lateral image was at the posterior upper edge of the S1 body. After placing the working cannula under the endoscope, the dural sac and epidural fat could be revealed. After the epidural fat was treated with radiofrequency, the dural sac, nerve root, and protruding disc tissue could be exposed. Rotation of the working cannula was used to avoid the dural sac inwardly and the nerve root outwardly while removing the protruding disc tissue. After complete hemostasis, the channel and the light source were gradually withdrawn, and the closing of the ligamentum flavum could be observed. After removal of the working cannula, the skin was sutured with one stitch and covered with a sterile dressing. One to three days after the surgery, the patient was discharged wearing a girdle.

### Postoperative evaluation

Postoperative evaluations included the straight leg raise test, sensory motor function assessments of both lower extremities, and other neurological examinations. Before being discharged, the patients received CT and MRI examinations of the same segment to assess the decompression. VAS scores were used to evaluate preoperative and postoperative leg pain, and ODI scores were used to evaluate preoperative and postoperative self-care ability in daily life; for long-term efficacy, the modified Macnab standard was used [7]. Telephone or outpatient follow-up was conducted at 1, 6, 12 and 48 months after discharge. The VAS and ODI scores were recorded at every follow-up.

### Statistical analysis

Continuous numerical variables with normal distribution are expressed as x ± std., and single-factor repeated measures variance analysis was used for comparisons between multiple groups. *P* < 0.05 was considered to indicate significant differences.

## Results

**Fig. 4:**
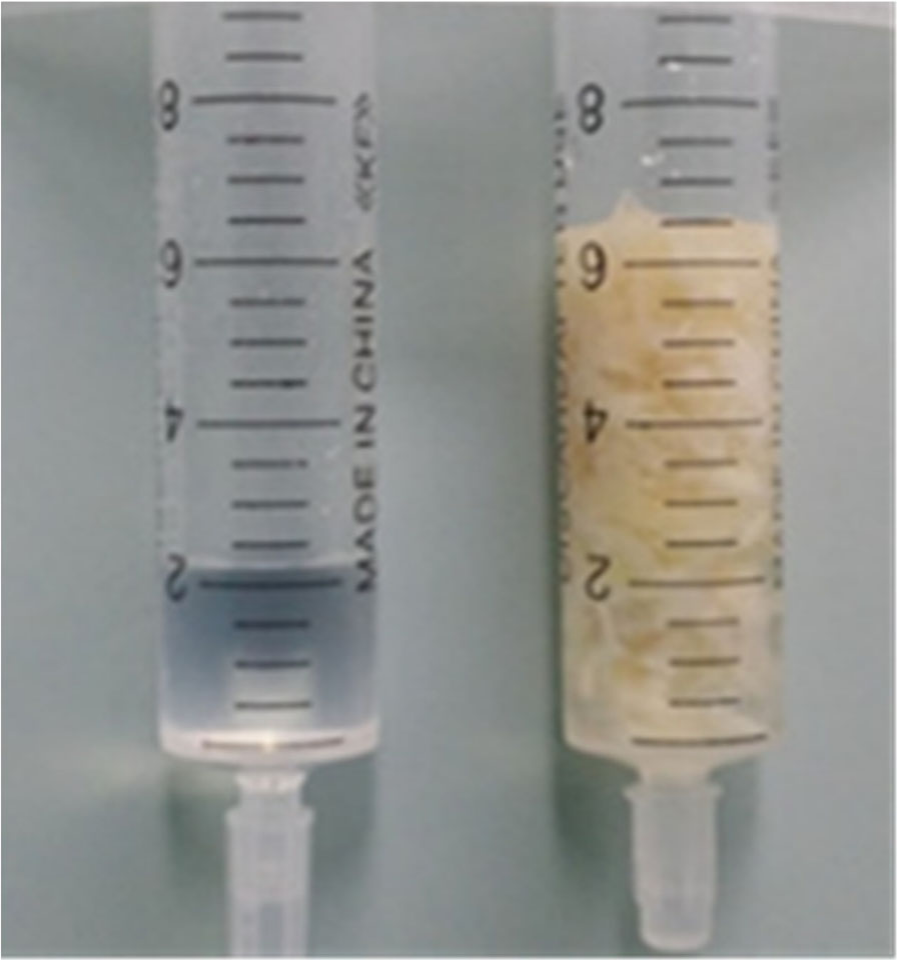
Specimens taken intraoperatively were measured by the volumetric method

Ninety-eight patients successfully completed the surgery; the operation time was 50–140 min, with an average of 90 min. Intraoperative bleeding was minimal but could not be accurately measured due to the continuous rinsing with saline. The amount of the nucleus pulposus removed was measured by the volumetric method (Fig. 4) and ranged from 2 to 4 ml, with an average of 2.8 ml. The postoperative hospitalization time was 1–4 days (average 2.8 d). The clinical results are shown in Table 2. One case showed dural sac injury because the puncture guide needle was inserted too deeply; however, since the diameter of the guide needle was 1.2 mm, there was no significant cerebrospinal fluid leakage after the needle was removed. Seven patients experienced feelings of numbness in the dermatome areas of the corresponding dominating nerve roots; this may be associated with the excessive power of the intraoperative radiofrequency, but there was no movement disorder and no significant root pain, and the symptoms improved significantly after oral administration of mecobalamin and aescuven forte for two weeks. There was no motor dysfunction, infection, hematoma, intestinal injury, or other complications. All patients were negative on the postoperative straight leg raise test. The postoperative MRI review results showed that the protruding disc tissue was almost completely resected in all patients, and there was no significant residue of protruding disc tissue. One patient showed a segment positioning error due to the lumbarization of the sacrum; intraoperative repositioning was performed, and the surgery was completed. Two obese female patients reported recurrent back and leg pain at the third and fourteen months, respectively, after the surgery, and MRI indicated disc herniation. The former patient chose classic posterior decompression fusion and internal fixation, while the latter chose PPFED. The remaining 96 cases were followed up for four years, and there were no recurrences of disc herniation; the patients returned to normal social and work activities, and there was no occurrence of secondary lumbar instability. The postoperative VAS and ODI scores were significantly improved compared with the preoperative scores (*P* < 0.05), see Table 1. During the last follow-up, the modified Macnab standard was used to assess efficacy; 92.6% of the cases were rated as excellent or good (Table 2).

**Table 2:** Clinical results and treatment effect at last follow-up

Demographics	Number of patients (%)
Clinical results	
Dural sac injury	1 (1.06%)
Nerve root outer membrane damage	1 (1.06%)
Sensory numbness of lower limbs	7 (7.45%)
Motor dysfunction	0 (0%)
Recurrent back and leg pain	2 (2.13%)
Reoperation	2 (2.13%)
Infection	0 (0%)
Cerebrospinal fluid leakage	0 (0%)
Other complications	0 (0%)
Rating	
Excellent	67 (71.3%)
Good	20 (21.3%)
Fair	7 (7.4%)
Poor	0 (0%)

## Discussion

The success rate of surgical treatment of LDH is 82–95.8% [3, 6]. The efficacy of treatment depends primarily on the cases selected; there have been no obvious associations with the choice of surgical technique [8, 9]. The selection of appropriate cases and the application of an endoscopic technique can greatly reduce the injury to normal tissues, optimizing the treatment efficacy of LDH [3, 10]. This procedure was developed from the percutaneous transforaminal endoscopic discectomy technique under local anesthesia to treat LDH, with endoscopic resection of the protruding intervertebral disc via various approaches under general anesthesia according to the patient’s requirements for pain management. The spinal full-endoscopic technique has become a minimally invasive and effective option for the ladder treatment of lumbar degenerative diseases [3, 5, 7]. Due to the influence of multiple anatomical structural particularities in the lumbosacral region, including high iliac ridge, small articular process hyperplasia, transverse process hypertrophy, transverse process space stenosis and many other factors, the treatment of L5 - S1 LDH by the full-endoscopic transforaminal technique is obviously limited [3, 5, 6]. At the same time, it is more difficult to separate the adhesions between calcified foci and nerve roots under the endoscope [6, 9, 11]. The modified transiliac approach full-endoscopic technique has a definite therapeutic effect, but the operation is more complex and difficult to master, making it more difficult for beginners to learn [11, 12]. However, the interlaminar approach is more in line with the surgical habits of surgeons and can fully expose the lesions in the spinal canal [8, 9]. The surgical field is relatively clear, and the range of exploration is wide. The protruding disc tissues and calcification foci can be fully removed, and the nerve roots can be separated to achieve sufficient decompression [9, 10].

The interlaminar space of the L5 - S1 segment is the most prone to the occurrence of LDH [3, 6]. Because its interlaminar space is relatively wider, it has anatomical advantages for the posterior approach for spinal endoscopic discectomy [9, 13]. The interlaminar approach of full-endoscopic surgery is more in line with the routine surgical path, and the procedure that targets the spinal protruding disc tissue is also safe [9, 14]. The lumbar 5 lamina is tilted at a 5- to 10-degree downward and backward angle in its coronal plane, which is not perpendicular to the upper lamina [15]. The lower edge of the lamina can be seen to block the interlaminar space in the anteroposterior image. Ebraheim et al. [16] analyzed the position of intervertebral discs on corpses and found that the L5 - S1 spinal canal only accommodates the dural sac and the sacral nerve root; its spatial structure is more spacious. The exits of the S1 nerve root are mainly on the head side of the interlaminar space of L5 - S1. The departure angle of the S1 nerve root exiting the dural sac is 18–26 degrees; most of the roots cross the intervertebral disc, which is the anatomical basis for removing the protruding disc tissue from the axilla of the nerve root [16]. Different location puncture techniques were selected according to the location of disc protrusion; this can be achieved using the shortest distance from the body surface to the lesion and targeted removal of the prominent interdisc organization. If the disc of L5 - S1 protrudes on the shoulder of the nerve root, the compressed S1 nerve root migrates inward, creating more operation space. In most patients, the protruding disc tissue is located at the axilla of the nerve root, which increases the departure angle of the nerve root exiting the dural sac and creates a space for grafting the working cannula under conditions that do not damage the nerve root. For protruding disc tissue on the shoulder of the nerve root, the nerve root migrates inward and downward, and it is also easy to manipulate the working cannula in the shoulder area. When the protruding disc tissue is completely located on the ventral side of the nerve root and shows severe compression, the nerve root is used as a center for grafting the cannula. The opening of the cannula faces the spinous process, and under X-ray, the position of the tip of the cannula does not exceed the middle of the pedicle. The epidural fat is the first structure that enters the vision and is easy to identify. After radiofrequency treatment, the dural sac and nerve root can be clearly exposed. The protruding disc tissue can then also be identified from the shoulder and the axilla of the nerve root, the head or the tail of the cannula is tilted appropriately to complete the removal of the disc from the shoulder or axilla of the nerve root so that the S1 nerve root obtains protection under direct vision, and the thoroughness of decompression is evaluated [17]. For large intervertebral disc herniations, the tissue structure in the spinal canal under the endoscope may be confusing. When reading the preoperative CT or MRI imaging data, attention should be paid in advance. If the nerve root is difficult to identify when it is severely compressed, a surgical probe can first be used to identify the outer edge of the dural sac and can then be moved forward to identify the intervertebral disc space, thereby identifying the protruding disc tissue or nerve root. Alternatively, an appropriate amount of ligamentum flavum or a small amount of bone tissue can be removed along the medial margin of the articular process to expand the lateral recess and make it possible to identify the nerve root; following this, a surgical probe can be used to probe and identify the protruding disc tissue and interlaminar space along the shoulder or axilla of the nerve root. The cannula and the light source need to be intraoperatively adjusted in a timely manner to appropriate positions according to the need for removal of the disc tissue, and various types of nucleus pulposus clamps are used to fully remove the protruding disc tissue. Radiofrequency is used to treat intraoperative bleeding to fully maintain a clear surgical field. Continuous saline rinsing can avoid heat damage to the nerve root from the radiofrequency treatment so that the disc tissue can be safely removed to achieve effective decompression of the S1 nerve root [8, 10, 11].

The ligamentum flavum in the interlaminar space of L5 - S1 is the thinnest of all the interlaminar spaces; it ranges in thickness from 2 to 6 mm [10, 18]. We used the cannula to break through the ligamentum flavum layer by layer rather than cutting it into the posterior wall of the spinal canal. This procedure ensures that the opening of the ligamentum flavum can be closed naturally when the surgery is completed; this helps restore the barrier between the epidural cavity and the muscle tissue outside the spinal canal and reduces the chances of fibrous scar tissue formation [19]. Even if an open surgery is performed later, the anatomical layers of the ligamentum flavum are easy to identify [19].

The completion of interlaminar full-endoscopic surgery under low-concentration continuous epidural anesthesia avoids the insufficient analgesia of local anesthesia, which can cause the patient to experience intraoperative pain or anxiety. Low-concentration anesthetics only block the sensations of the lower limbs while preserving motor sensations and are suitable for patients with other medical diseases and for patients who are at high risk for complications during general anesthesia [20]. Some patients cannot achieve epidural grafting tube anesthesia due to spinal degeneration or may voluntarily choose general anesthesia. In the early exploratory study, one of the patients in the study cases in this group suffered an injury to the outer membrane of the nerve root. This injury was caused by accident when a nucleus pulposus clamp was used to take a large piece of disc tissue, a complication that was not adequately realized preoperatively.

This study has some limitations. First, it is a single-center preoperative and postoperative self-controlled study of a small sample; there is no surgical control group. Second, the technique used in this study requires additional expensive professional equipment and involves the exposure of both the patient and the physician to radiation. Moreover, in terms of a learning curve for practitioners with rich experience in open surgery, the interlaminar endoscopic technique is easier to master than the posterolateral transforaminal endoscopic technique. However, the first 10 surgeries required the instruction of an experienced superior physician [9, 21]. When facing the endoscope, the surgeon must know the head, tail, medial side, and lateral side of the spinal canal and when to use a nucleus pulposus clamp, a laminectomy punch, radiofrequency, and other equipment; the surgeon also needs to clearly recognize the anatomical positions reached [9, 19, 21, 22]. As long as the intraoperative procedure is performed gently and carefully and the anatomical structures are clearly identified, the step-by-step removal of the protruding disc tissue is safe.

## Conclusions

In summary, the treatment of L5 - S1 intervertebral disc herniation with PPFED by grafting tubes at various positions via an interlaminar approach is a safe, effective, and minimally invasive surgical method. Reaching the location of disc herniation directly through the natural gap of the bones can maximally avoid collateral injury from spinal surgery. Compared with other related clinical reports, this study achieved similar clinical treatment results.

## Data Availability

The data generated and analyzed during this study are included in this published article and in its supplementary information files.

## Abbreviations

LDH: Lumbar disc herniation
ODI: Oswestry disability index
PPFED: Posterior percutaneous full-endoscopic discectomy
VAS: Visual analog scale
